# Numerical Integration of the Master Equation in Some Models of Stochastic Epidemiology

**DOI:** 10.1371/journal.pone.0036160

**Published:** 2012-05-02

**Authors:** Garrett Jenkinson, John Goutsias

**Affiliations:** Whitaker Biomedical Engineering Institute, The Johns Hopkins University, Baltimore, Maryland, United States of America; University of Leeds, United Kingdom

## Abstract

The processes by which disease spreads in a population of individuals are inherently stochastic. The master equation has proven to be a useful tool for modeling such processes. Unfortunately, solving the master equation analytically is possible only in limited cases (e.g., when the model is linear), and thus numerical procedures or approximation methods must be employed. Available approximation methods, such as the system size expansion method of van Kampen, may fail to provide reliable solutions, whereas current numerical approaches can induce appreciable computational cost. In this paper, we propose a new numerical technique for solving the master equation. Our method is based on a more informative stochastic process than the population process commonly used in the literature. By exploiting the structure of the master equation governing this process, we develop a novel technique for calculating the *exact* solution of the master equation – up to a desired precision – in certain models of stochastic epidemiology. We demonstrate the potential of our method by solving the master equation associated with the stochastic SIR epidemic model. MATLAB software that implements the methods discussed in this paper is freely available as Supporting Information S1.

## Introduction

Stochasticity can play an important role when studying a disease that spreads through a population of individuals [1]–[3]. A common approach to modeling this problem is by means of a Markov process, whose probability distribution satisfies a deterministic differential equation known as the master equation. Solving the master equation analytically however is not in general possible and Monte Carlo sampling, based on the Gillespie algorithm [4], is often used to accomplish this goal. Unfortunately, accurate evaluation of the probability distribution of a Markov process requires a prohibitively large number of Monte Carlo samples for most systems of interest. As a consequence, Monte Carlo sampling is mostly used to estimate statistical summaries of the underlying stochastic population dynamics, such as means and variances.

To evaluate the solution of the master equation, a number of approximation techniques have been proposed in the literature, such as the system-size expansion method of van Kampen [1], [5], [6]. While approximations may work well in certain circumstances, they often fail when the underlying assumptions are not satisfied. The system-size expansion method for example can only produce a normal approximation to the solution of the master equation. Therefore, if the probability distribution of the population process is bimodal, then this method will produce erroneous results.

Some effort has recently shifted away from Monte Carlo sampling and approximation techniques and has focused on exploiting the linear structure of the master equation associated with the population process. This results in a numerical solution to the master equation through matrix exponentiation; e.g., see [2], [7]–[10]. A popular technique along these lines employs a Krylov subspace approximation (KSA) method [7], [8] that dramatically reduces the size of matrix exponentiation and results in an attractive iterative algorithm for solving the master equation. However, the KSA technique is based on several approximations, whose cumulative effect may appreciably affect the method's accuracy, numerical stability, and computational efficiency.

There are two main issues that can affect performance of the KSA method. One is choosing the dimension of the approximating Krylov subspace used. If the dimension is chosen too small, the method may produce an inaccurate solution to the master equation, whereas, a value that is too large can result in an appreciable decrease of computational efficiency. Unfortunately, there is no rigorous way to optimally determine an appropriate value for this parameter, which is chosen manually, even in advanced implementations such as Expokit [7]. Another issue is the fact that, at each step, the KSA method may not necessarily produce a probability vector (i.e., a vector composed of nonnegative elements that sum to one). This problem can be addressed by using a sufficiently small step-size, but this may seriously affect the method's computational efficiency. In practice, the KSA method is equipped with a heuristic step that zeros-out all negative values and re-normalizes the positive values so that they sum to one. This step however introduces its own errors, which may affect the quality of the approximation in a manner that is not easy to predict.

Instead of using the population process, we can describe the stochastic spread of a disease by a more informative stochastic process known as the degree-of-advancement (DA). Exploiting the structure of the master equation governing this process results in a novel numerical algorithm for calculating the *exact* solution of the master equation, up to a desired precision, which we refer to as the implicit Euler (IE) method. This technique enjoys several advantages over the KSA method: its global error is of first-order with respect to the step-size, it is numerically stable regardless of the step-size used, and always produces a solution whose elements are nonnegative and sum to one. As we will discuss in this paper, the IE method shows great promise for solving certain problems in stochastic epidemiology in which the sample space associated with the DA process is reasonably sized. It is not however meant to replace the KSA method, which is still the best numerical method available for solving the master equation in problems where implementation of the IE method is not computationally attractive or possible. To illustrate the potential of the proposed IE method, we calculate the solution of the master equation associated with the stochastic SIR epidemic model and use this solution to study some important properties of this model.

## Methods

### Disease dynamics

The classical SIR epidemic model (without births, deaths, or imports of disease) is one of the simplest models in epidemiology [11]. Here, each individual in a population is either susceptible to a disease, infected, or recovered. If we denote by 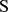, 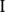, and 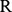 the susceptible, infected and recovered individuals, respectively, and by 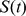, 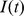 and 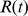 their corresponding (and possibly random) population numbers, we can characterize the state of the SIR model at time 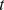 by using the 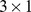 vector 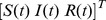, where 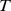 denotes vector transpose. The state depends on time due to the (possibly random) occurrences of the following two reactions:
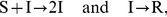
(1)which model infection of a susceptible individual (first reaction) as well as recovery of an infected individual (second reaction).

We can model a complex epidemiological system in more general terms by using the following reactions:
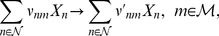
(2)where 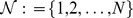 and 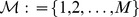. This model congregates individuals into 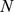 different groups, 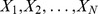, which interact through 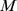 coupled reactions. Parameters 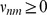 and 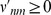 are the *stoichiometry coefficients* of the 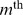 reaction. These parameters tell us how individuals interact with each other as well as their status after occurrence of a particular reaction. For example, in the aforementioned SIR model, we may set 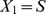, 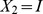, 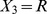, resulting in 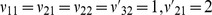, with the remaining coefficients being zero.

The usual way to characterize an epidemiological system is by means of the 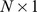 random vector 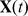 with elements 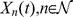, where 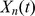 denotes the population of the 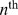 group of individuals present in the system at time 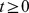. By convention, we set 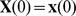, for some known value 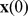 (i.e., we assume that we know the initial population numbers at time 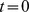). We refer to the multivariate stochastic process 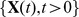 as the *population process*.

Let 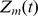 be the (possibly random) number of times that the 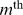 reaction occurs during the time interval 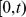 and 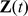 be the 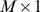 random vector with elements 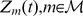. Then, 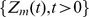 is a counting process, known as the *degree of advancement* (DA) of the 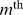 reaction [5]. We set 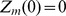 and refer to the multivariate stochastic process 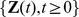 as the *DA process*. Note that
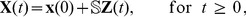
(3)where 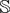 is the 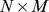 *net* stoichiometry matrix of the system with elements 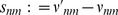. Therefore, and for a given initial population vector 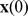, equation (3) allows us to uniquely determine the population process 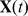 from the DA process 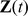. However, we cannot in general determine the DA process from the population process. This can only be done when the nullity of 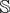 is zero, in which case 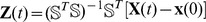. As a consequence, the DA process is more informative than the population process.

### The master equation

To model an epidemiological system governed by the reactions in (2), we must specify, for each 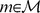, the probability that one reaction 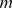 will occur within an infinitesimally small time interval 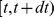. For most systems of interest, this probability is given by 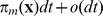, where 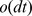 is a term that goes to zero faster than 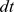 and 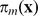 is a (usually nonlinear) function of individual populations at time 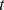, known as the *propensity function* of the 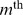 reaction [12]. It turns out that the DA process 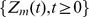 is a Markovian counting process with intensity 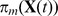. As a result, it can be shown that the probability mass function 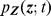 of the DA process satisfies the following master equation [13], [14]:
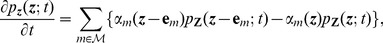
(4)for 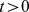, initialized by 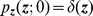, with 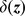 being the Kronecker delta function, where
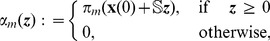
(5)and 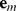 is the 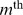 column of the 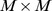 identity matrix. In the theory of Markov processes, the master equation is a special case of the more general forward Kolmogorov equation [5].

We can use the solution 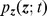 of the previous master equation to calculate the probability mass function 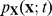 of the population process. Since we are dealing with discrete random variables, this calculation is straightforward:
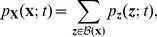
(6)where 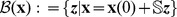. Therefore, by solving the master equation (4) [i.e., by calculating the probability mass function 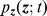, for 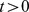], we can completely specify the dynamic properties of a Markovian model. However, and for most cases of interest, this is a notoriously difficult task, both analytically and computationally. In the following, we propose a promising numerical method to address this problem and illustrate its potential using a simple example based on the stochastic SIR epidemic model.

### Exploiting structure

Most available algorithms for solving the master equation focus on the population process instead of the DA process. It turns out that, by using the DA process, we may reap some benefits that can lead to a simple numerical solver for the general master equation (4).

In the following, we assume that statistical analysis of an epidemiological model of interest is limited within a finite time interval 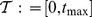, where 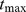 is the maximum time for which the DA process is almost surely contained within an 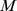-dimensional discrete and finite sample space 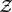; i.e.,
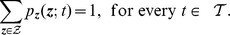
We index the elements in 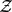 by 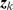, 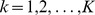, where 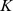 is the cardinality of 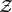 (i.e., the total number of elements in 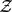). We can then define the 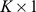 vector 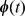 with elements 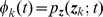, for 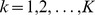. Clearly, 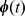 specifies the probability mass function 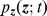. It can be seen from (4) that 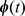 can be calculated by solving the following system of 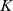 *linear* ordinary differential equations (ODEs):
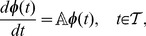
(7)where 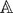 is a 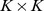 matrix that can be directly constructed from the master equation. In the theory of Markov processes, 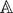 is known as the *generator matrix*. Note that the 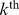 column of 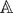 contains zeros in most places except for the 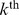 element that takes value 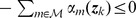 and 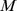 off-diagonal elements that take values 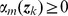, 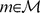. Therefore, the elements of each column of 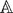 add to zero. Note also that equation (7) is initialized by a vector 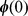 whose first element equals 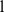 (assuming that 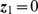), whereas, the remaining elements are all zero.

The main advantage of using the DA process 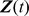 is that, under an appropriate ordering of the elements in 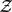, the generator matrix 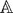 will be *lower triangular*. We will shortly demonstrate that this can result in substantial simplification of the numerical algorithm used to solve (7).

To obtain a matrix 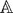 that is lower triangular, we must order the points 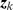 in the sample space 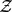 *lexicographically*, such that 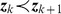, for 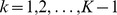, where 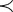 denotes that one variable is lexicographically smaller than another [e.g., 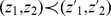 if and only if 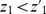 or 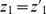 and 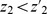]. Because a reaction can only *increase* (by one) the value of a single element of 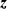, it is not possible for probability mass to be transferred from 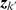 to 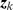 when 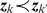. Such monotonic transfer of probability does not generally occur when the population process 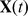 is used. Therefore, when the points 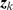, 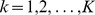, in 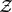 are ordered lexicographically, the 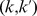 element of matrix 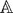 will be zero when 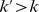 and, therefore, 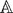 will be lower triangular. An example is provided in Supporting Information S2.

### Numerical solver

We now proceed by exploiting the three key structural characteristics of matrix 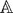: its stability, triangularity, and sparsity. We have noted that the diagonal elements of 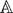 are non-positive. However, since 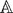 is triangular, its diagonal elements will be the eigenvalues of 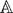. Thus, the linear constant coefficient system of ODEs given by (7) is *stable*, ensuring the efficacy of implicit ODE solvers [15]. As a consequence, we can use the implicit Euler method to estimate 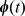 at discrete time points 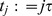, 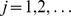, for a given time step 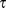. Then, given an estimate 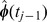 of 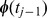, we can obtain an estimate 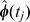 of 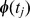 by solving the following system of linear equations:
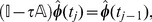
(8)where 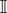 is the 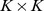 identity matrix. By initializing this computation with 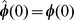, we can therefore recursively calculate the values of the probability mass function 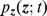 of the DA process at the discrete time points 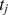, 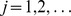. In Supporting Information S2, we show that solving the previous system is always possible, for any 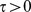, due to the invertibility of matrix 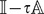. We also show that this procedure always returns a probability vector for any step-size 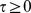. Moreover, we demonstrate that the resulting method is a first-order solver, since the *global* error 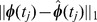 is of 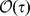 (i.e., the global error is proportional to the step-size 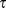). Finally, since the implicit Euler step is always stable for *any* choice of 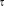 [15], the errors from previous iterations will not be amplified in later stages, regardless of the step-size used. Therefore, a desired error can be achieved by simply reducing the value of the step-size 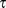. We refer to the resulting technique for solving the master equation based on (8) as the *implicit Euler* (IE) method.

In general, solving (8) would require 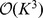 computations, where 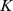 is the cardinality of the sample space 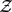, which will be prohibitive. However, since 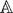 is a *triangular* matrix, we can use forward substitution whose cost is usually of 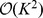. But since 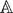 is a *sparse* matrix, with each column having only 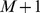 non-zero elements, forward substitution can be done at a cost of 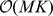 [16], where 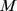 is the number of reactions. In addition, calculating the probability mass function at time 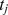 requires storage of 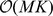 nonzero numbers. In particular, we need to store 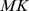 nonzero elements of matrix 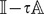 as well as 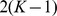 elements of vectors 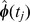 and 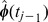 [note that the elements of each column of matrix 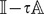 and the elements of each of the two vectors 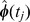 and 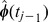 sum to one]. Since 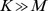, the computational and memory requirements of the IE method will be 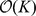, which grow *linearly* in terms of 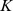.

### Matrix exponentiation

Instead of the previous approach, we may attempt to solve the master equation governing the population process 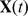 by a matrix exponentiation method [2], [9]. Let 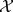 be an 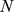-dimensional discrete and finite sample space such that
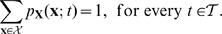
If we index the elements in 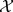 by 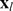, 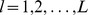, where 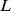 is the cardinality of 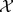, then we can define the 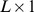 vector 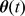 with elements 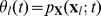, for 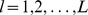. In this case, the probability mass function 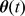 can be calculated by solving the following system of 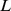 *linear* ODEs:
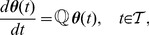
(9)where 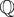 is a sparse 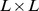 matrix whose structure can be inferred directly from the master equation [9]. Note that (9) is initialized by a vector 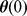 whose first element equals 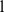 [assuming that 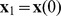], whereas, the remaining elements are all zero.

We can obtain estimates 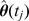 of 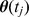, for 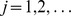, by using the following recursion:
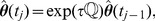
(10)initialized with 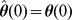. The main issue with this equation however is the need to evaluate the matrix exponential 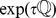. Although many techniques are currently available to do this job, they are not very satisfactory due to issues related to stability, accuracy, and computational efficiency [17].

The best method currently available to compute (10) is based on a *Krylov subspace approximation* (KSA) method [7], [8], [18]. In its simplest form, the method approximates 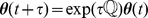, for a small 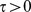, by 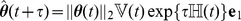, where 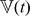 is an 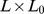 matrix whose columns form an orthonormal basis for the 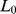-dimensional Krylov subspace 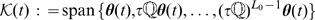, 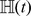 is an 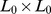 matrix computed during the well-known Arnoldi procedure used to calculate 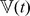, and 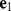 is the first column of the 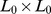 identity matrix. Then, the value of 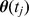 is recursively approximated by
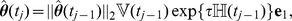
for 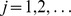, with 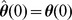. The KSA method reduces the problem of calculating the exponential of the large and sparse 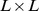 matrix 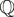 to the problem of calculating the exponential of the much smaller and *dense* 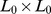 matrix 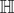 (note that 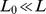, with 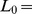
30–50 being sufficient for most applications). Computation of the reduced size problem can be done by standard methods, such as Chebyshev or Padé approximation [7], [8], [17].

As we mentioned in the Introduction, there are several disadvantages of using the KSA method: possible error accumulation that may lead to instabilities, inability to produce, at each step, a probability vector without heuristically modifying the calculated values, and a need to specify an appropriate dimensionality for the Krylov subspace without appreciably affecting computational efficiency while achieving acceptable numerical accuracy. These issues are nicely circumvented by the IE method, but with a price: the proposed method can be applied to a smaller set of problems than the KSA method.

### Practical considerations

In general, the computational and memory requirements of matrix exponentiation grow *quadratically* in terms of the cardinality 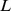 of the sample space 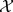, and can quickly become prohibitive for large values of 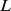. The KSA method however can greatly reduce this expense to 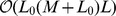 computations and 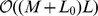 memory locations, where 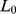 is the dimension of the approximating Krylov subspace used and 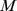 is the number of reactions; see our discussion in Supporting Information S2. Thus, the relative efficiency of the IE method, which requires 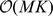 computation and storage cost, to the KSA approach will depend on the relative values of the cardinalities 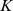 and 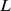 of the sample spaces 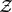 and 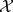, respectively.

As we mentioned before, if the nullity of the net stoichiometry matrix 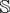 is zero, then there is a one-to-one correspondence between 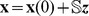 and 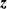. As a consequence of (6), the cardinalities of 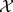 and 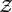 will be the same, in which case 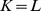. Under these circumstances, the IE method will outperform the KSA method. This is a consequence of the fact that 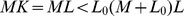 and 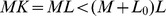 in this case. We can easily verify that, for the simple SI model (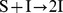), the SIR epidemic model characterized by (1), and the SEIR model (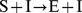, 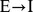, 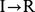, where E denotes a group of individuals exposed to disease but not yet infectious), the nullity of 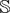 is indeed zero and, therefore, the IE method will be superior to the KSA method.

In general, the IE method will be computationally superior to the KSA method, provided that the cardinality of the sample space 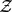 is not appreciably larger than 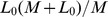 times the cardinality of the sample space 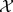 [or not much larger than 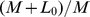 times the cardinality of the sample space 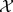, if we also consider memory requirements]. Of course, in situations where the nullity of 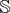 is large, the sample space 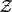 can become appreciably larger than 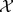, in which case the KSA method will be more preferable. Note that there are cases in which 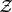 and 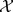 can become infinite (e.g., suppose an influx of people at some constant rate 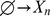, in which case both sample spaces will be unbounded). In these situations, the use of a finite state projection approach [9] is required to reduce the sample spaces, and the relative efficiency of the two methods will depend on the sizes of the resulting subspaces.

For a given step-size 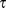, the IE method described so far generates a sequence of probability vectors 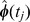, 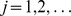. Assuming that the true solution 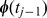 is known at time 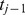, we can show [see equation (S.11) in Supporting Information S2] that the *local* error 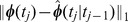 is of 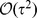, where 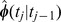 is the approximation of 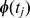 obtained by the IE method for a given value of 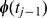. We can further improve this result by employing a powerful computational tool known as Richardson extrapolation [19].

**Figure 1:**
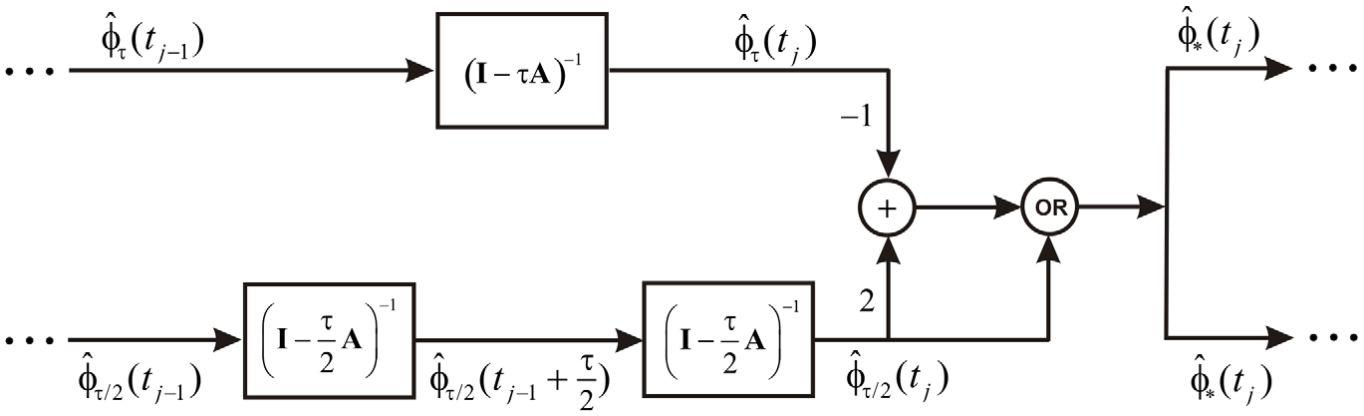
One step of the RIE method. The upper branch implements the standard IE method with step-size 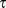, whereas, the lower branch implements the IE method with step-size 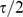. “OR” implements equation (11).

We have shown in the Supporting Information S2 that, if 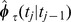 and 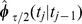 are the approximations of 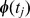 obtained from 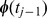 by the IE method with step-sizes 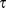 and 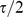, respectively, then 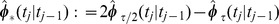 also approximates 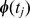, but with a local error of 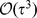. We therefore expect that 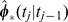 is a better approximation to 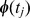 than 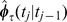 [or even 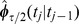; see Supporting Information S2] for a sufficiently small step-size 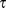. This suggests that we can use a valuable modification of the IE method to obtain a better approximation to the solution of the master equation than the original technique. This modification combines two runs of the IE method, with time steps 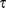 and 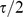, and produces a solution 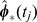, given by
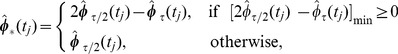
(11)where 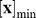 denotes the minimum value of the elements of vector 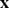. In this case, 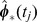 is given by the ‘improved’ vector 
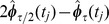
 only when all elements of that vector are nonnegative. Otherwise, 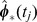 is given by the vector 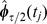 calculated by the IE method with the smaller step-size 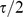. This assures that 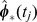 is always a probability vector. We will be referring to the resulting technique as the Richardson-based implicit Euler (RIE) method. We illustrate one step of this method in Figure 1.

Many ODE solvers, including the KSA method, adjust the step-size at each iteration to assure that the local error 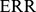 is less than a pre-specified error tolerance 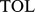 while minimizing the computational effort required to accomplish this goal. We can also modify the RIE method to accommodate variable step-sizes. By following our analysis in the Supporting Information S2, we can approximately calculate the local error 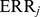 at step 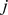 by [see equation (S.16)]
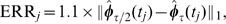
where we use a factor of 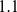 to compensate for the possibility that the true (but unknown) local error is larger (by 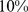) than the actual error calculated by 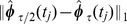. If 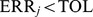, then we consider the step successful and increase the step-size from 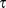 to 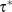, where [see equation (S.17) in Supporting Information S2]
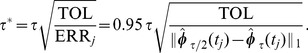
(12)However, if 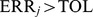, then the step is unsuccessful. In this case, we decrease the step-size from 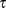 to 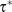 by using (12) and redo the RIE step.

We finally note that some readers might be concerned with precision loss in the forward substitution step of the IE and RIE methods. To address this issue, we could employ the standard numerical technique of *iterative improvement* [15] with moderate additional computational cost. However, we show in the Supporting Information S2 that the matrix 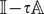 being inverted is never singular. Moreover, this matrix is far from being singular (i.e., its eigenvalues are not numerically close to zero) as 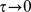. We therefore suggest that a preferable method of combating precision loss is to reduce 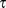, since the step-size tightly regulates the global error as well (see Supporting Information S2). Although in the subsequent example we did not perform iterative improvement, the results indicate that any precision loss is negligible, despite the large dynamic range of probability values involved in the solution.

## Results

To demonstrate the efficacy of our method, we tackle the problem of modeling a well-documented 1978 influenza epidemic in an English boarding school [20]. A deterministic SIR model was originally developed to analyze these data [21]. Subsequently, the model was extended to the stochastic case and approximately solved using van Kampen's system-size expansion method [1]. In the following, we use the IE method to compute the *exact* solution of the underlying master equation up to a desired precision.

There are three classes of individuals, 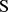, 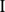 and 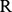, representing 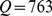 susceptible, infected and recovered pupils. Spreading of the epidemic is governed by the reactions in (1) with propensity functions
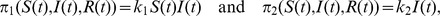
where 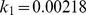/day and 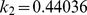/day are the rate constants of infection and recovery, respectively [1]. The initial conditions are given by
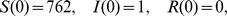
reflecting the fact that only one pupil is infected at the start of the epidemic. We take the sample space 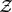 to be the rectangular region in the 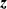 plane that begins at 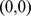 and extends to include the maximal point 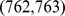. This is due to the fact that the first reaction can occur at most 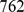 times, after which all pupils will have been infected, whereas, the second reaction can occur at most 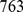 times, after which all pupils will have recovered from the infection. As a consequence, the sample space 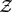 contains 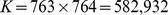 points.

Numerically solving the master equation over a period of 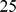 days by means of the KSA method using Expokit [7] took 72 minutes of CPU time on a 2.20 GHz Intel Mobile Core 2 Duo T7500 processor running Matlab 7.7. The resulting solution produces an L2 error 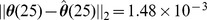, where 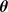 is a solution of the master equation obtained by a stringent run of Expokit, which we consider to be the ‘true’ solution. The required 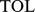 value (used to determine a desired error tolerance for the KSA method and for the RIE method with variable step-size) was set to 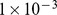. We obtained the Expokit solution by using a Krylov subspace approximation with dimension 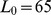. This value was determined by starting with the default value of 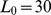 and successively increasing it by 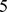 until the resulting Expokit error estimate was less than 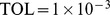. The reported L2 errors were calculated using a solution obtained by a computationally more expensive Expokit run with 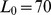 and 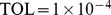, which we consider it to be the ‘true’ solution. This is based on the premise that Expokit will produce the true solution for sufficiently large 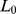 and small 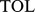.

To be compatible with Expokit, we report here the L2 error. Note however that the error analysis of our method, provided in the Supporting Information S2, is based on the L1 error. On the other hand, using equation (8) with 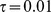 days, the IE method took a mere 53 seconds of CPU time, achieving a smaller (by a factor of 2.8) final L2 error of 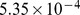. We can achieve a further reduction of the L2 error by using the RIE method with fixed step-size. This is clear from the results summarized in Table 1. This performance can be achieved however at the expense of increasing the CPU time required to calculate the solution. Note that we may be able to decrease the CPU time by using the RIE method with variable step-size (see Table 1). This method however results in a noticeable decrease of accuracy (at least for the example considered here), with an L2 error that is 2.8 times larger than the one obtained with the KSA method.

**Table 1:** The L2 error and the CPU time associated with the four numerical methods discussed in this paper.

Numerical Method	L2 Error	CPU Time
KSA	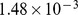	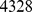 seconds
IE	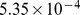	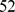 seconds
RIE (fixed step-size)	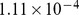	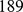 seconds
RIE (variable step-size)	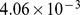	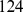 seconds

**Figure 2:**
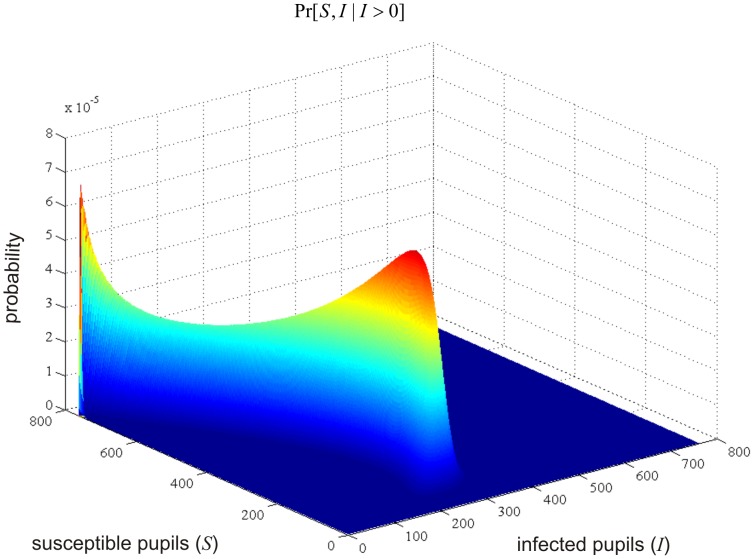
A snapshot of the calculated probability mass function. Joint conditional probability mass function 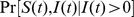 of susceptible and infected pupils at the end of the 6th day of the influenza epidemic.

Since 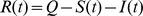, it suffices to focus on the joint probability mass function 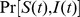 of susceptible and infected pupils. It turns out however that the epidemic-free state occurs with high probability 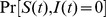, a situation that visually obscures the values of 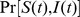. For this reason, instead of 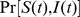, we depict in Figure 2 a snapshot of the calculated joint conditional probability mass function 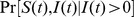 of the susceptible and infected pupils at the end of the 6th day, given that at least one pupil is infected. The Supporting Information S3 contains a .gif movie that depicts the dynamic evolutions of 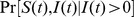 and 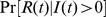 during the 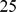 day period. We have obtained these and all subsequent results by exclusively using the basic IE method.

**Figure 3:**
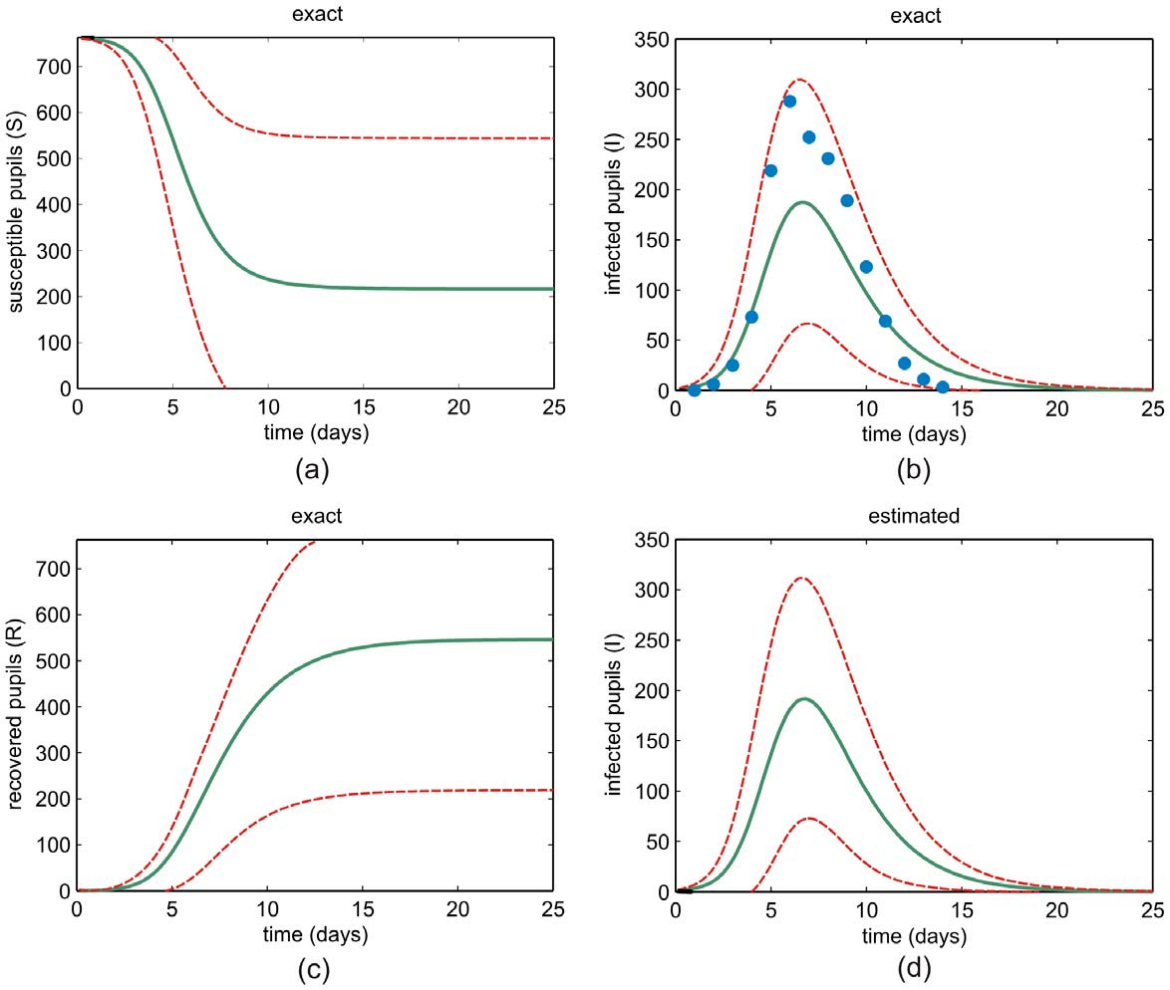
Dynamic mean and standard deviation profiles. The mean profiles (solid green lines) and the 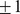 standard deviation profiles (dashed red lines) of: (a) susceptible, (b) infected, and (c) recovered pupils. Monte Carlo estimates of the mean and standard deviation profiles of the infected pupils are depicted in (d). Blue circles in (b) mark available data [20].

In Figure 3, we depict the dynamic profiles of the mean numbers of susceptible, infected and recovered pupils (solid green lines) as well as the dynamic profiles of the 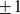 standard deviations (dashed red lines), computed directly from the joint probability mass function 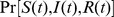. We also depict the observed data (blue circles) obtained from the literature [20]. These results are identical to the results obtained by Monte Carlo estimation based on 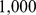 trajectories sampled from the master equation using the Gillespie algorithm (only data related to the infected pupils are shown), and assures that the IE method produces the correct results. Unfortunately, we cannot employ the Gillespie algorithm to accurately estimate the joint probability mass function 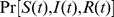 in a reasonable time, due to the prohibitively large number of samples required by this method.

The bimodal nature of the probability mass function depicted in Figure 2 clearly demonstrates that the system-size expansion method used previously in [1] is not appropriate for this model, since the method leads to a unimodal Gaussian approximation. As a matter of fact, the results depicted in Figure 3 are different than the mean and standard deviation profiles depicted in Figures 3–4 of [1]. Because of the Gaussian nature of the system-size expansion method, the results reported in [1] over-estimate the means and under-estimate the standard deviations, since this technique is blind to the bimodal nature of the probability distribution. As a matter of fact, using the means and standard deviations to characterize the stochastic properties of individual classes in the SIR model is not appropriate. This is also evident by the fact that the 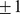 standard deviations can take negative values as well as values greater than 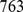. In Figure 3, we have truncated these misleading values.

**Figure 4:**
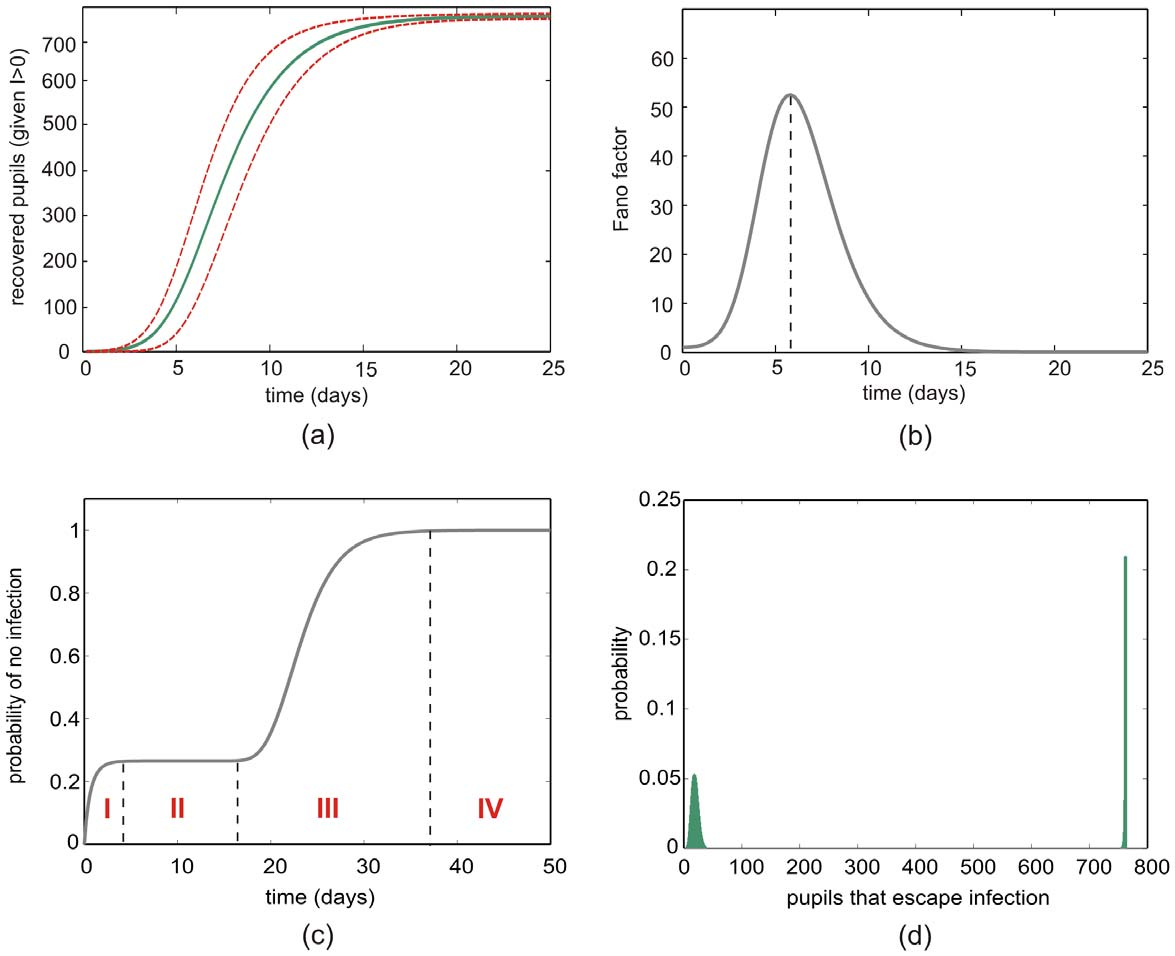
Dynamic properties of the SIR model. (a) Evolution of the expected number of recovered pupils (solid green line) and the 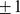 standard deviations (dashed red lines), given that at least one pupil is always infected. (b) The Fano factor (variance/mean) associated with the results in (a) as a function of time. (c) Dynamic evolution of the probability of extinction 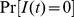, 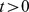. (d) The (approximately stationary) probability mass function 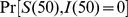 at 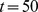 days.

We can use the calculated joint probability mass functions 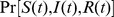 to study a number of dynamic properties of the SIR model in a stochastic setting. In Figure 4(a), for example, we depict the evolution of the expected number of recovered pupils (solid green line), as well as the 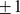 standard deviations (dashed red lines), given that at least one pupil is always infected. During the first few days, few infections occur, and the expected number of recovered pupils will almost be zero. Subsequently, this number increases monotonically to 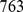, following a near sigmoidal curve. The 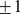 standard deviation curves and the evolution of the Fano factor (variance/mean) depicted in Figure 4(b) indicate that there is appreciable fluctuation in the number of recovered pupils during days 3–10, after which most pupils recover from the infection. According to the results depicted in Figure 4(b), the maximum fluctuation in the number of recovered pupils occurs during the 6th day.

In Figure 4(c), we depict the dynamic evolution of the calculated probability of extinction 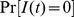, 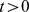, during a period of 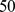 days. This evolution is characterized by four phases. During phase I (days 1–4), the probability of extinction increases rapidly from 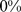 to about 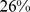, due to the small number of infectious pupils. During phase II (days 5–17), the probability remains relatively constant to about 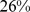. During this period of time, the epidemic takes its natural course, increasingly infecting susceptible individuals, who eventually recover from the disease. As a consequence, we do not expect the probability of extinction to increase during this phase. On the other hand, during phase III (days 18–40), the number of infected pupils monotonically decreases to zero. It is therefore expected that, during this phase, the probability of extinction will monotonically increase to its maximum value of one. Finally, during phase IV (days 40–50), there is no infectious pupils present. As a result, the influenza virus cannot be transmitted to the remaining susceptible pupils and the epidemic ceases to exist.

When studying an epidemic model with extinction, a task of practical interest is to calculate the number of individuals that escape infection. This is usually done by evaluating the expected number 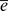 of individuals that escape infection (or the average number of susceptible individuals that remain after extinction) as the mean value of the stationary probability mass function 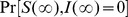 [2]. In Figure 4(d), we depict the joint probability 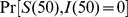 at time 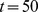 days, which we assume to be a very close approximation to the stationary probability mass function 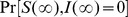. By using this probability, we compute 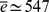. Note however that, due to the bimodal nature of 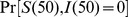, calculating 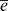 is misleading. On the other hand, by using the result depicted in Figure 4(d), we can confirm that there is a 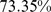 chance that 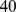 pupils or less, and a 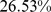 chance that 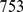 pupils or more, escape infection. Clearly, these ‘confidence intervals’ provide a more accurate statistical assessment of the number of individuals that escape infection than 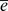. Interestingly, there is only 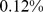 chance that the number of pupils escaping infection is within the range 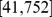, which includes the value of 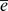.

## Discussion

Modeling the stochastic dynamics of a disease that spreads through a small and well-mixed population of individuals is an increasingly important subject of modern epidemiology. Unfortunately, even for the simplest model, calculating the underlying probability distribution is a daunting task.

In an effort to address this problem, we have introduced in this paper a new approach to numerically compute the probability mass function of a Markovian population process governed by the master equation. Implementation of this approach is feasible when the number of possible states is not prohibitively large. In this case, the proposed method can lead to *exact* statistical analysis – up to a desired precision – of certain stochastic epidemiological models of interest, such as the SIR epidemic model.

The method introduced in this paper is linear – both in terms of memory and computational requirements – with respect to the cardinality 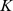 of the sample space 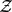 of the degrees of advancement of the underlying reactions. As a consequence, the method is feasible any time 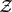 is relatively small. In general, however, the cardinality of 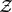 may grow arbitrarily large, making implementation of the method impossible without an appropriate FSP approximation [9]. Thus, the proposed technique is only applicable to models that constrain the number of reaction events, such as the SIR epidemic model considered in this paper, or models for which the number of reaction events is sufficiently small during a time period of interest (i.e., models without ‘fast’ reactions). Moreover, due to the well-known problem of the ‘curse of dimensionality,’ 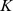 grows exponentially with respect to the number of reactions 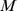. Hence, models with many reactions cannot be solved by the proposed method.

An effort is currently underway to reduce the size of the sample space 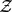, without compromising accuracy. A plausible way to accomplish this goal is to reduce the number of reactions involved by removing ‘fast’ reactions using a multi-scale approximation technique, such as one of the techniques introduced for biochemical reaction systems [13], [14], [22], and to adaptively update 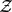 at each time point 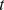 by confining it to the smallest possible subspace 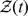 of 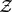. Because of the lower-triangular and sparse nature of matrix 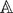 in (8), it is also plausible that we employ optimized algorithms developed for solving sparse triangular systems of linear equations on parallel and distributed memory computer architectures [23], indicating that future efforts towards solving the master equation could potentially focus on using high-performance computing systems.

Finally, it was brought to our attention by one of the reviewers that, in an earlier work, K. N. Crank proposed a method to map a general Markovian population process on a countable sample space to an augmented Markovian process with triangular generator matrix [24] by appropriately ordering that space. Crank's technique can be easily combined with our implicit Euler method to construct an alternative algorithm for numerically solving the master equation with focus on the population process instead of the DA process. However, we cannot find any advantage of using Crank's approach over ours. We believe that an approach for numerically solving the master equation based on the DA process is more preferable than an approach based on the population process. The former can provide the probability distributions of both the DA and population processes, whereas, the latter can only produce the probability distribution of the population process. Moreover, the IE method based on the DA process is easier to implement, due primarily to a faster and more natural implementation of the lexicographic ordering used by this approach as opposed to the more complex ordering of the population sample space proposed by Crank. For more details on this issue, see our discussion in Supporting Information S2.

## Supporting Information

Supporting Information S1 This file contains the MATLAB code used to generate the results presented in the paper.
(ZIP)

Supporting Information S2 This file contains additional information and proofs that elucidate various mathematical and numerical aspects of the work presented in the paper. It also provides a brief discussion of an alternative method for solving the master equation using the implicit Euler method, based on ordering the population sample space instead of the DA sample space.
(PDF)

Supporting Information S3 This file contains a video of the dynamic evolution of the joint conditional probability mass function of susceptible and infected pupils in an influenza epidemic predicted by the SIR model.
(GIF)
