# Supplementary figures and images for: Numerical Integration of the Master Equation in Some Models of Stochastic Epidemiology
Source: PLoS One. 2012 May 2;7(5):e36160. doi: 10.1371/journal.pone.0036160 (PMC3342242; doi:10.1371/journal.pone.0036160)

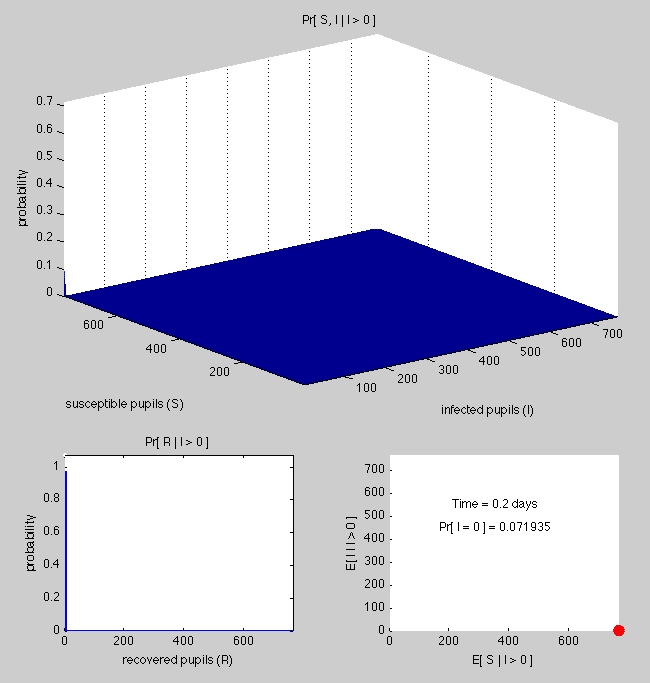

Supplement: Supporting Information S1 — This file contains the MATLAB code used to generate the results presented in the paper. (ZIP) [file pone.0036160.s001.zip › matlab/MakeFigures/results/SIR.gif]
