# Supplementary material for: Numerical Integration of the Master Equation in Some Models of Stochastic Epidemiology
Source: PLoS One. 2012 May 2;7(5):e36160. doi: 10.1371/journal.pone.0036160 (PMC3342242; doi:10.1371/journal.pone.0036160)
Supplement: Supporting Information S2 — This file contains additional information and proofs that elucidate various mathematical and numerical aspects of the work presented in the paper. It also provides a brief discussion of an alternative method for solving the master equation using the implicit Euler method, based on ordering the population sample space instead of the DA sample space. (PDF) [file pone.0036160.s002.pdf]

Supporting Information S2

for "Numerical integration of the master equation in some models of stochastic epidemiology"

Garrett Jenkinson and John Goutsias
Whitaker Biomedical Engineering Institute, The Johns Hopkins University, Baltimore, MD 21218, USA
E-mail: Garrett Jenkinson – jenkinson@jhu.edu; John Goutsias – goutsias@jhu.edu

Triangularization of the generator matrix $\mathbb{A}$

In this section, we use a simple example to illustrate why lexicographic ordering of the elements of the sample space $\mathscr{Z}$ leads to a lower triangular generator matrix $\mathbb{A}$ in equation (7) of the main text.

Let us consider the SIR model and denote by Z_1, Z_2 the DAs of the two reactions $\mathrm{S} + \mathrm{I} \to 2\mathrm{I}$ and $\mathrm{I} \to \mathrm{R}$, respectively. We will assume that, initially, there are two susceptible individuals, one infected individual, and no recovered individuals; i.e., we will assume that $x_1(0) = 2$, $x_2(0) = 1$, and $x_3(0) = 0$. This implies that $0 \leq Z_1(t) \leq 2$ and $0 \leq Z_2(t) \leq 3$, at any time $t > 0$, which is due to the fact that the first reaction will occur at most two times, after which all individuals will be infected, whereas, the second reaction can occur at most three times, after which all individuals will recover from the infection. In this case, lexicographic ordering of the elements of the two-dimensional sample space $\mathscr{Z}$ results in the following twelve points:

$$
\begin{array}{ll}
\boldsymbol{z}_1 = (0,0) & \boldsymbol{z}_2 = (0,1) \\
\boldsymbol{z}_3 = (0,2) & \boldsymbol{z}_4 = (0,3) \\
\boldsymbol{z}_5 = (1,0) & \boldsymbol{z}_6 = (1,1) \\
\boldsymbol{z}_7 = (1,2) & \boldsymbol{z}_8 = (1,3) \\
\boldsymbol{z}_9 = (2,0) & \boldsymbol{z}_{10} = (2,1) \\
\boldsymbol{z}_{11} = (2,2) & \boldsymbol{z}_{12} = (2,3).
\end{array}
$$

As a consequence, the probability vector $\boldsymbol{\phi}(t)$ in equation (7) of the main text is given by

$$
\boldsymbol{\phi}(t) = \begin{pmatrix}
\Pr[Z_1(t) = 0, Z_2(t) = 0] \\
\Pr[Z_1(t) = 0, Z_2(t) = 1] \\
\Pr[Z_1(t) = 0, Z_2(t) = 2] \\
\Pr[Z_1(t) = 0, Z_2(t) = 3] \\
\Pr[Z_1(t) = 1, Z_2(t) = 0] \\
\Pr[Z_1(t) = 1, Z_2(t) = 1] \\
\Pr[Z_1(t) = 1, Z_2(t) = 2] \\
\Pr[Z_1(t) = 1, Z_2(t) = 3] \\
\Pr[Z_1(t) = 2, Z_2(t) = 0] \\
\Pr[Z_1(t) = 2, Z_2(t) = 1] \\
\Pr[Z_1(t) = 2, Z_2(t) = 2] \\
\Pr[Z_1(t) = 2, Z_2(t) = 3]
\end{pmatrix}. \tag{S.1}
$$

Let us now assume that the propensity functions of the two SIR reactions are given by

$$
\begin{aligned}
\pi_1(x_1, x_2, x_3) &= k_1 x_1 x_2 \\
\pi_2(x_1, x_2, x_3) &= k_2 x_2,
\end{aligned}
$$

where k_1 and k_2 are two rate constants and x_1, x_2, and x_3 denote the number of susceptible, infected, and recovered individuals, respectively. Since $x_1(0) = 2$, $x_2(0) = 1$ and $x_3(0) = 0$, equation (3) of the

main text implies that

$$
\begin{aligned}
X_1(t) &= 2 - Z_1(t) \\
X_2(t) &= 1 + Z_1(t) - Z_2(t) \\
X_3(t) &= Z_2(t),
\end{aligned}
$$

which, together with equation (5) of the main text, results in

$$
\begin{aligned}
\alpha_1(z_1, z_2) &= k_1(2 - z_1)(1 + z_1 - z_2) \\
\alpha_2(z_1, z_2) &= k_2(1 + z_1 - z_2),
\end{aligned} \tag{S.2}
$$

for $0 \leq z_1 \leq 2$ and $z_2 \leq 1 + z_1$, whereas, $\alpha_1(z_1, z_2) = \alpha_2(z_1, z_2) = 0$, otherwise. As a consequence of equations (4) and (7) of the main text and (S.1), (S.2), the generator matrix $\mathbb{A}$ is given by

$$
\mathbb{A} = \begin{pmatrix}
-(2k_1 + k_2) & 0 & 0 & 0 & 0 & 0 & 0 & 0 & 0 & 0 & 0 & 0 \\
k_2 & 0 & 0 & 0 & 0 & 0 & 0 & 0 & 0 & 0 & 0 & 0 \\
0 & 0 & 0 & 0 & 0 & 0 & 0 & 0 & 0 & 0 & 0 & 0 \\
0 & 0 & 0 & 0 & 0 & 0 & 0 & 0 & 0 & 0 & 0 & 0 \\
2k_1 & 0 & 0 & 0 & -2(k_1 + k_2) & 0 & 0 & 0 & 0 & 0 & 0 & 0 \\
0 & 0 & 0 & 0 & 2k_2 & -(k_1 + k_2) & 0 & 0 & 0 & 0 & 0 & 0 \\
0 & 0 & 0 & 0 & 0 & k_2 & 0 & 0 & 0 & 0 & 0 & 0 \\
0 & 0 & 0 & 0 & 0 & 0 & 0 & 0 & 0 & 0 & 0 & 0 \\
0 & 0 & 0 & 0 & 2k_1 & 0 & 0 & 0 & -3k_2 & 0 & 0 & 0 \\
0 & 0 & 0 & 0 & 0 & k_1 & 0 & 0 & 3k_2 & -2k_2 & 0 & 0 \\
0 & 0 & 0 & 0 & 0 & 0 & 0 & 0 & 0 & 2k_2 & -k_2 & 0 \\
0 & 0 & 0 & 0 & 0 & 0 & 0 & 0 & 0 & 0 & k_2 & 0
\end{pmatrix},
$$

which is indeed sparse and lower triangular. Note that states that cannot occur are assigned zero propensities. These states correspond to the zero rows in $\mathbb{A}$ [e.g., this is true for state $(0, 2)$, which is associated with the third row of $\mathbb{A}$ and would result in a negative number of -1 infected individuals]. Note also that the non-zero diagonal elements of this matrix are all negative, with the remaining nonzero elements being positive. Finally, each column of $\mathbb{A}$ sums to zero.

Invertibility of matrix $\mathbb{I} - \tau\mathbb{A}$

We will show that matrix $\mathbb{B} := \mathbb{I} - \tau\mathbb{A}$ is *invertible*, for any $\tau > 0$. Indeed, for each column k of $\mathbb{B}$, the element b_{kk} is strictly greater than the sum of the absolute values of the remaining elements $b_{k'k}$, $k' \neq k$, since

$$
b_{kk} = 1 - \tau a_{kk} = 1 + \tau \sum_{m \in \mathcal{M}} \alpha_m(\boldsymbol{z}_k) > \tau \sum_{m \in \mathcal{M}} \alpha_m(\boldsymbol{z}_k) = \tau \sum_{k' \neq k} a_{k'k} = \sum_{k' \neq k} |b_{k'k}|,
$$

for $\tau > 0$, by virtue of the fact that $a_{kk} = -\sum_{m \in \mathcal{M}} \alpha_m(\boldsymbol{z}_k)$ and $a_{k'k} = \alpha_m(\boldsymbol{z}_k)$, whenever $\boldsymbol{z}_{k'} = \boldsymbol{z}_k + \boldsymbol{e}_m$, where $\boldsymbol{e}_m$ is the mth column of the $M \times M$ identity matrix, and $a_{k'k} = 0$, otherwise. Thus, $\mathbb{B}$ is invertible according to Theorem 6.1.10 in [1].

The IE method produces a probability vector

We will now show that, at each iteration j, the IE method produces a probability vector $\widehat{\boldsymbol{\phi}}(t_j)$ for any step-size τ [i.e., all elements of $\widehat{\boldsymbol{\phi}}(t_j)$ are nonnegative and sum to one]. Since the initial vector $\widehat{\boldsymbol{\phi}}(0)$ is

taken to be a probability vector, we must only show that, if $\widehat{\boldsymbol{\phi}}(t_{j-1})$ is a probability vector, then $\widehat{\boldsymbol{\phi}}(t_j)$ is a probability vector as well.

We will first show that

$$\widehat{\phi}_k(t_{j-1}) \geq 0 \implies \widehat{\phi}_k(t_j) \geq 0, \quad \text{for every } k = 1, 2, \ldots, K, \tag{S.3}$$

where $\widehat{\phi}_k(t_{j-1})$ and $\widehat{\phi}_k(t_j)$ are the kth elements of $\widehat{\boldsymbol{\phi}}(t_{j-1})$ and $\widehat{\boldsymbol{\phi}}(t_j)$, respectively. Note that the off-diagonal elements of matrix $\mathbb{B} := \mathbb{I} - \tau\mathbb{A}$ are nonpositive, since $b_{k'k} = -\tau a_{k'k} = -\tau\alpha_m(\boldsymbol{z}_k) \leq 0$, for $k' \neq k$. Furthermore, using the same argument as in the previous section, we can show that $\mathbb{B} + t\mathbb{I}$ is nonsingular for every $t \geq 0$. According to Theorem 2.5.3 in [2], all elements of matrix $\mathbb{B}^{-1}$ are nonnegative. Since $\widehat{\boldsymbol{\phi}}(t_j) = \mathbb{B}^{-1}\widehat{\boldsymbol{\phi}}(t_{j-1})$, we obtain (S.3).

We will now show that

$$\mathbf{1}^T\widehat{\boldsymbol{\phi}}(t_{j-1}) = 1 \implies \mathbf{1}^T\widehat{\boldsymbol{\phi}}(t_j) = 1,$$

where the elements of vector $\mathbf{1}$ are all equal to one. Indeed, we have that

$$\begin{aligned} 1 &= \mathbf{1}^T\widehat{\boldsymbol{\phi}}(t_{j-1}) \\ &= \mathbf{1}^T(\mathbb{I} - \tau\mathbb{A})\widehat{\boldsymbol{\phi}}(t_j) \\ &= \mathbf{1}^T\widehat{\boldsymbol{\phi}}(t_j) - \tau\mathbf{1}^T\mathbb{A}\widehat{\boldsymbol{\phi}}(t_j) \\ &= \mathbf{1}^T\widehat{\boldsymbol{\phi}}(t_j) - \tau\mathbf{0}^T\widehat{\boldsymbol{\phi}}(t_j) \\ &= \mathbf{1}^T\widehat{\boldsymbol{\phi}}(t_j), \end{aligned} \tag{S.4}$$

where the elements of vector $\mathbf{0}$ are all equal to zero. The second equality in (S.4) comes from equation (8) of the main text, whereas, the fourth equality comes from the fact that the elements of each column of matrix $\mathbb{A}$ sum to zero.

Note that the previous arguments do not depend on the particular value of the step-size τ. Hence, $\widehat{\boldsymbol{\phi}}(t_j)$ is a probability vector for any value of τ.

The global error of the IE method is of $\mathcal{O}(\tau)$

In this section, we show that the global error $||\boldsymbol{\phi}(t_j) - \widehat{\boldsymbol{\phi}}(t_j)||_1$ associated with the IE method, where $t_j := j\tau$, is of $\mathcal{O}(\tau)$.

Note that $\boldsymbol{\phi}(t_j) = \exp(\tau\mathbb{A})\boldsymbol{\phi}(t_{j-1})$ and $(\mathbb{I} - \tau\mathbb{A})\widehat{\boldsymbol{\phi}}(t_j) = \widehat{\boldsymbol{\phi}}(t_{j-1})$. Thus,

$$\boldsymbol{\phi}(t_j) = \exp(\tau\mathbb{A})\boldsymbol{\phi}(t_{j-1}) = \cdots = \exp(j\tau\mathbb{A})\boldsymbol{\phi}(0)$$

$$\widehat{\boldsymbol{\phi}}(t_j) = (\mathbb{I} - \tau\mathbb{A})^{-1}\widehat{\boldsymbol{\phi}}(t_{j-1}) = \cdots = (\mathbb{I} - \tau\mathbb{A})^{-j}\widehat{\boldsymbol{\phi}}(0).$$

As a result,

$$\exp(-j\tau\mathbb{A})\boldsymbol{\phi}(t_j) = (\mathbb{I} - \tau\mathbb{A})^j\widehat{\boldsymbol{\phi}}(t_j), \tag{S.5}$$

since $\widehat{\boldsymbol{\phi}}(0) = \boldsymbol{\phi}(0)$. However,

$$\exp(-\tau\mathbb{A}) = \mathbb{I} - \tau\mathbb{A} + \mathcal{O}(\tau^2). \tag{S.6}$$

From (S.5) and (S.6), we have that

$$\exp(-j\tau\mathbb{A})\boldsymbol{\phi}(t_j) = \left[\exp(-\tau\mathbb{A}) - \mathcal{O}(\tau^2)\right]^j\widehat{\boldsymbol{\phi}}(t_j) = \left[\exp(-j\tau\mathbb{A}) - j\mathcal{O}(\tau^2)\right]\widehat{\boldsymbol{\phi}}(t_j).$$

Consequently,

$$\exp(-j\tau\mathbb{A})[\boldsymbol{\phi}(t_j) - \widehat{\boldsymbol{\phi}}(t_j)] = -j\mathcal{O}(\tau^2)\widehat{\boldsymbol{\phi}}(t_j) \iff \boldsymbol{\phi}(t_j) - \widehat{\boldsymbol{\phi}}(t_j) = -j\mathcal{O}(\tau^2)\exp(j\tau\mathbb{A})\widehat{\boldsymbol{\phi}}(t_j),$$

which implies that

$$\begin{aligned}||\boldsymbol{\phi}(t_j) - \widehat{\boldsymbol{\phi}}(t_j)||_1 &= ||j\mathcal{O}(\tau^2)\exp(j\tau\mathbb{A})\widehat{\boldsymbol{\phi}}(t_j)||_1 \\ &\leq j\mathcal{O}(\tau^2)\,||\exp(j\tau\mathbb{A})||_1||\widehat{\boldsymbol{\phi}}(t_j)||_1 \\ &= j\mathcal{O}(\tau^2) \\ &= \frac{t_j}{\tau}\,\mathcal{O}(\tau^2) \\ &\leq \frac{t_{\max}}{\tau}\,\mathcal{O}(\tau^2), \end{aligned} \tag{S.7}$$

where $t_{\max}$ is the maximum simulation time. To obtain (S.7), we have used the fact that $||\exp(j\tau\mathbb{A})||_1 = 1$, since $\mathbb{A}$ is the generator matrix of a Markov process, and $||\widehat{\boldsymbol{\phi}}(t_j)||_1 = 1$. As a result, we finally obtain $||\boldsymbol{\phi}(t_j) - \widehat{\boldsymbol{\phi}}(t_j)||_1 \leq t_{\max}\mathcal{O}(\tau)$, which implies that $||\boldsymbol{\phi}(t_j) - \widehat{\boldsymbol{\phi}}(t_j)||_1 = \mathcal{O}(\tau)$.

Computational and storage requirements of KSA method

The Arnoldi procedure performed at each step of the KSA method requires L_0 matrix-vector multiplications between matrix $\mathbb{Q}$ and the probability distribution $\boldsymbol{\theta}$, resulting in a cost of $\mathcal{O}(L_0 L^2)$ computations in general. However, the sparsity of $\mathbb{Q}$ [matrix $\mathbb{Q}$ has $(M+1)L$ non-zero elements instead of L^2] reduces this cost to $\mathcal{O}(L_0(M+1)L)$. Additionally, the orthonormalization step in the Arnoldi procedure requires $\mathcal{O}(L_0^2 L)$ operations due to inner product computations. Finally, the Krylov subspace approximation step requires that matrix $\mathbb{V}$ is multiplied with the first column of the matrix exponential $\exp(\tau\mathbb{H})$, at a cost of $\mathcal{O}(L_0 L)$. By summing these costs, we can see that the total computational cost of the KSA method is of $\mathcal{O}(L_0(M+L_0)L)$. On the other hand, the storage requirements are of $\mathcal{O}((M+L_0)L)$, where $\mathcal{O}(ML)$ memory locations are required for storing $\mathbb{Q}$ and $\mathcal{O}(L_0 L)$ locations are required for storing matrix $\mathbb{V}$, which is multiplied with the first column of the matrix exponential $\exp(\tau\mathbb{H})$.

Justification of Richardson extrapolation

To justify the Richardson extrapolation procedure used to improve the accuracy of the IE method, let us assume that the solution $\boldsymbol{\phi}(t_{j-1})$ of equation (7) of the main text is known at time t_{j-1}. Then, the approximate solution $\widehat{\boldsymbol{\phi}}(t_j \mid t_{j-1})$ obtained by the IE method at time t_j satisfies

$$\widehat{\boldsymbol{\phi}}(t_j \mid t_{j-1}) = \boldsymbol{\phi}(t_{j-1}) + \tau\mathbb{A}\widehat{\boldsymbol{\phi}}(t_j \mid t_{j-1}), \tag{S.8}$$

by virtue of equation (8) of the main text. We now have that

$$\begin{aligned}\boldsymbol{\phi}(t_j) - \widehat{\boldsymbol{\phi}}(t_j \mid t_{j-1}) &= \boldsymbol{\phi}(t_j) - \boldsymbol{\phi}(t_{j-1}) - \tau\mathbb{A}\widehat{\boldsymbol{\phi}}(t_j \mid t_{j-1}) \\ &= \boldsymbol{\phi}(t_j) - \boldsymbol{\phi}(t_{j-1}) - \tau\mathbb{A}\boldsymbol{\phi}(t_{j-1}) - \tau^2\mathbb{A}^2\widehat{\boldsymbol{\phi}}(t_j \mid t_{j-1}) \\ &= \boldsymbol{\phi}(t_j) - \boldsymbol{\phi}(t_{j-1}) - \tau\mathbb{A}\boldsymbol{\phi}(t_{j-1}) - \tau^2\mathbb{A}^2\boldsymbol{\phi}(t_{j-1}) + \mathcal{O}(\tau^3), \end{aligned} \tag{S.9}$$

where we have used (S.8) twice. A Taylor series expansion of $\boldsymbol{\phi}(t_{j-1}+\tau)$ around t_{j-1} gives

$$\begin{aligned}\boldsymbol{\phi}(t_j) &= \boldsymbol{\phi}(t_{j-1}+\tau) \\ &= \boldsymbol{\phi}(t_{j-1}) + \tau\frac{d\boldsymbol{\phi}(t_{j-1})}{dt} + \frac{1}{2}\,\tau^2\frac{d^2\boldsymbol{\phi}(t_{j-1})}{dt^2} + \mathcal{O}(\tau^3) \\ &= \boldsymbol{\phi}(t_{j-1}) + \tau\mathbb{A}\boldsymbol{\phi}(t_{j-1}) + \frac{1}{2}\,\tau^2\mathbb{A}^2\boldsymbol{\phi}(t_{j-1}) + \mathcal{O}(\tau^3), \end{aligned} \tag{S.10}$$

by virtue of equation (7) of the main text, which, together with (S.9), results in

$$\boldsymbol{\phi}(t_j) = \widehat{\boldsymbol{\phi}}_\tau(t_j \mid t_{j-1}) - \frac{1}{2}\,\tau^2 \mathbb{A}^2 \boldsymbol{\phi}(t_{j-1}) + \mathcal{O}(\tau^3), \tag{S.11}$$

where we now use $\widehat{\boldsymbol{\phi}}_\tau(t_j \mid t_{j-1})$ to denote the fact that the approximate solution $\widehat{\boldsymbol{\phi}}(t_j \mid t_{j-1})$ is obtained with step-size τ.

Let us now denote by $\widehat{\boldsymbol{\phi}}_{\tau/2}(t_j \mid t_{j-1})$ the approximate solution obtained by the IE method at time t_j when the step-size is $\tau/2$. Note that $\widehat{\boldsymbol{\phi}}_{\tau/2}(t_{j-1} + \tau/2 \mid t_{j-1}) = (\mathbb{I} - \tau\mathbb{A}/2)^{-1}\boldsymbol{\phi}(t_{j-1})$ and $\widehat{\boldsymbol{\phi}}_{\tau/2}(t_j \mid t_{j-1}) = (\mathbb{I} - \tau\mathbb{A}/2)^{-1}\widehat{\boldsymbol{\phi}}_{\tau/2}(t_{j-1} + \tau/2 \mid t_{j-1})$, by virtue of equation (8) of the main text. Therefore, $\widehat{\boldsymbol{\phi}}_{\tau/2}(t_j \mid t_{j-1}) = (\mathbb{I} - \tau\mathbb{A}/2)^{-2}\boldsymbol{\phi}(t_{j-1})$, or [compare with (S.8)]

$$\widehat{\boldsymbol{\phi}}_{\tau/2}(t_j \mid t_{j-1}) = \boldsymbol{\phi}(t_{j-1}) + \tau\mathbb{A}\widehat{\boldsymbol{\phi}}_{\tau/2}(t_j \mid t_{j-1}) - \frac{\tau^2}{4}\mathbb{A}^2\widehat{\boldsymbol{\phi}}_{\tau/2}(t_j \mid t_{j-1}). \tag{S.12}$$

We now have that

$$\begin{aligned}
\boldsymbol{\phi}(t_j) - \widehat{\boldsymbol{\phi}}_{\tau/2}(t_j \mid t_{j-1}) &= \boldsymbol{\phi}(t_j) - \boldsymbol{\phi}(t_{j-1}) - \tau\mathbb{A}\widehat{\boldsymbol{\phi}}_{\tau/2}(t_j \mid t_{j-1}) + \frac{\tau^2}{4}\mathbb{A}^2\widehat{\boldsymbol{\phi}}_{\tau/2}(t_j \mid t_{j-1}) \\
&= \boldsymbol{\phi}(t_j) - \boldsymbol{\phi}(t_{j-1}) - \tau\mathbb{A}\boldsymbol{\phi}(t_{j-1}) + \frac{\tau^2}{4}\mathbb{A}^2\boldsymbol{\phi}(t_{j-1}) - \tau^2\mathbb{A}^2\widehat{\boldsymbol{\phi}}_{\tau/2}(t_j \mid t_{j-1}) + \mathcal{O}(\tau^3) \\
&= \boldsymbol{\phi}(t_j) - \boldsymbol{\phi}(t_{j-1}) - \tau\mathbb{A}\boldsymbol{\phi}(t_{j-1}) - \frac{3}{4}\,\tau^2\mathbb{A}^2\boldsymbol{\phi}(t_{j-1}) + \mathcal{O}(\tau^3),
\end{aligned} \tag{S.13}$$

where we have used (S.12) twice. From the Taylor series expansion (S.10) and (S.13), we finally obtain

$$\boldsymbol{\phi}(t_j) = \widehat{\boldsymbol{\phi}}_{\tau/2}(t_j \mid t_{j-1}) - \frac{1}{4}\,\tau^2\mathbb{A}^2\boldsymbol{\phi}(t_{j-1}) + \mathcal{O}(\tau^3). \tag{S.14}$$

Now, from (S.11) and (S.14), we have

$$\boldsymbol{\phi}(t_j) = 2\widehat{\boldsymbol{\phi}}_{\tau/2}(t_j \mid t_{j-1}) - \widehat{\boldsymbol{\phi}}_\tau(t_j \mid t_{j-1}) + \mathcal{O}(\tau^3). \tag{S.15}$$

This result shows that

$$\widehat{\boldsymbol{\phi}}_*(t_j \mid t_{j-1}) := 2\widehat{\boldsymbol{\phi}}_{\tau/2}(t_j \mid t_{j-1}) - \widehat{\boldsymbol{\phi}}_\tau(t_j \mid t_{j-1})$$

may produce a better approximation to $\boldsymbol{\phi}(t_j)$ than either $\widehat{\boldsymbol{\phi}}_\tau(t_j \mid t_{j-1})$ or $\widehat{\boldsymbol{\phi}}_{\tau/2}(t_j \mid t_{j-1})$, since it results in a third-order approximation (in terms of the local error) of $\boldsymbol{\phi}(t_j)$, as compared to $\widehat{\boldsymbol{\phi}}_\tau(t_j \mid t_{j-1})$ or $\widehat{\boldsymbol{\phi}}_{\tau/2}(t_j \mid t_{j-1})$ which result in second-order approximations.

We can also use $\widehat{\boldsymbol{\phi}}_\tau(t_j \mid t_{j-1})$ and $\widehat{\boldsymbol{\phi}}_{\tau/2}(t_j \mid t_{j-1})$ to determine an appropriate step-size τ_* that guarantees a local error within a pre-specified tolerance TOL. Indeed, if we define the local error ERR := $\|\boldsymbol{\phi}(t_j) - \widehat{\boldsymbol{\phi}}_{\tau/2}(t_j \mid t_{j-1})\|_1$, then from equation (S.15), we approximately have that

$$\mathrm{ERR} = \|\widehat{\boldsymbol{\phi}}_{\tau/2}(t_j \mid t_{j-1}) - \widehat{\boldsymbol{\phi}}_\tau(t_j \mid t_{j-1})\|_1, \tag{S.16}$$

which provides a way to calculate the error for a sufficiently small step-size τ. If now ERR $\neq$ TOL, then we need to change the step-size to a new value τ_*, such that $\boldsymbol{\phi}(t_j) - \widehat{\boldsymbol{\phi}}_{\tau_*/2}(t_j \mid t_{j-1}) = \boldsymbol{\phi}(t_j) - \widehat{\boldsymbol{\phi}}_{\tau/2}(t_j \mid t_{j-1})$, which will imply that TOL := $\|\boldsymbol{\phi}(t_j) - \widehat{\boldsymbol{\phi}}_{\tau_*/2}(t_j \mid t_{j-1})\|_1 = \|\boldsymbol{\phi}(t_j) - \widehat{\boldsymbol{\phi}}_{\tau/2}(t_j \mid t_{j-1})\|_1$ = ERR. From (S.14), we have that

$$\begin{aligned}
\boldsymbol{\phi}(t_j) - \widehat{\boldsymbol{\phi}}_{\tau/2}(t_j \mid t_{j-1}) &\simeq -\frac{1}{4}\,\tau^2\mathbb{A}^2\boldsymbol{\phi}(t_{j-1}) \\
\boldsymbol{\phi}(t_j) - \widehat{\boldsymbol{\phi}}_{\tau_*/2}(t_j \mid t_{j-1}) &\simeq -\frac{1}{4}\,\tau_*^2\mathbb{A}^2\boldsymbol{\phi}(t_{j-1}),
\end{aligned}$$

from which we obtain

$$\frac{\tau_*^2}{\tau^2} \simeq \frac{\|\boldsymbol{\phi}(t_j) - \widehat{\boldsymbol{\phi}}_{\tau_*/2}(t_j \mid t_{j-1})\|_1}{\|\boldsymbol{\phi}(t_j) - \widehat{\boldsymbol{\phi}}_{\tau/2}(t_j \mid t_{j-1})\|_1} = \frac{\mathrm{TOL}}{\mathrm{ERR}}\,.$$

As a consequence, the desired step-size value will approximately be given by

$$\tau^* = \tau\sqrt{\frac{\mathrm{TOL}}{\mathrm{ERR}}}\,. \tag{S.17}$$

An alternative ordering method

In [3], an ordering of the population sample space has been proposed that can be used to put equation (9)of the main text into a lower triangular form. The idea is to augment the population sample space $\mathcal{X}$ in a way that the augmented states can be ordered so that a reaction can only take a state from a smaller to a larger value with respect to this ordering. Then, by arranging the states of the augmented sample space in an increasing order, one ensures that the resulting generator matrix will be lower triangular.

To define such an ordering, the reaction system is augmented with an artificial 'counting' species X_{N+1}, whose value increases monotonically in a way which guarantees that the augmented population process $\{X_1(t), X_2(t), \ldots, X_{N+1}(t)\}$ will always be well-ordered. The augmented system is governed by the following M reactions [compare with equation (2) of the main text]

$$\sum_{n=1}^{N} \nu_{nm} X_n \to \sum_{n=1}^{N} \nu'_{nm} X_n + \nu'_{N+1,m} X_{N+1}, \quad m \in \mathcal{M},$$

whose propensity functions are the same as the original propensities. This implies that the DA dynamics will not change and, therefore, the joint probability distribution of $X_1(t), X_2(t), \ldots, X_N(t)$ can be calculated by marginalizing the joint probability distribution of the augmented states $X_1(t), X_2(t), \ldots, X_{N+1}(t)$ with respect to X_{N+1}.

The stoichiometry coefficient $\nu'_{N+1,m}$ must be chosen so that the reactions always move the state $\widetilde{\boldsymbol{X}} = \{X_1, X_2, \ldots, X_{N+1}\}$ from a lower value $\widetilde{\boldsymbol{x}}$ to a higher value $\widetilde{\boldsymbol{x}}' > \widetilde{\boldsymbol{x}}$ with respect to the following ordering rules:

1) $\sum_{n=1}^{N+1} x'_n > \sum_{n=1}^{N+1} x_n$, or

2) $\sum_{n=1}^{N+1} x'_n = \sum_{n=1}^{N+1} x_n$ and $x'_{N+1} > x_{N+1}$, or

3) $\sum_{n=1}^{N+1} x'_n = \sum_{n=1}^{N+1} x_n$, $x'_{N+1} = x_{N+1}$, and $x'_N > x_N$, or

⋮ ⋮

$N+1$) $\sum_{n=1}^{N+1} x'_n = \sum_{n=1}^{N+1} x_n$, $x'_{N+1} = x_{N+1}, \ldots, x'_3 = x_3$, and $x'_2 > x_2$.

Note that there are three types of reactions in the original system, given by equation (2) of the main text: (i) reactions for which $\sum_{n=1}^{N} s_{nm} > 0$, (ii) reactions for which $\sum_{n=1}^{N} s_{nm} = 0$, and (iii) reactions for which $\sum_{n=1}^{N} s_{nm} < 0$. If reaction m is of type (i), then $\nu'_{N+1,m}$ can be set equal to zero and the reaction will result in the state variables increasing according to rule 1. If a reaction m is of type (ii), then $\nu'_{N+1,m}$ can be set equal to one and this reaction will result in the state variables increasing according to rule 2. Finally, if reaction m is of type (iii), then $\nu'_{N+1,m}$ can be set equal to $-\sum_{n=1}^{N} s_{nm}$ and this reaction will

result in the state variables increasing according to rule 2. These observations lead to setting

$$\nu'_{N+1,m} := \left[\sum_{n=1}^{N} s_{nm} < 0\right] \left(-\sum_{n=1}^{N} s_{nm}\right) + \left[\sum_{n=1}^{N} s_{nm} = 0\right], \tag{S.18}$$

where $[\cdot]$ is the Iverson bracket that takes value one when its argument is true and zero otherwise.

The previous ordering can be used to construct an alternative numerical algorithm for solving the master equation of the population process using the implicit Euler method discussed in our paper. This algorithm however will not provide any obvious advantage over the method based on the DA process and, in particular, it will not resolve the important issue of the underlying sample space being unbounded. As a matter of fact, it is easy to see that if the artificial 'counting' population process $X_{N+1}(t)$ is almost surely bounded, then the DA process will be almost surely bounded as well. Moreover, if the DA process is almost surely unbounded and X_n is almost surely bounded, for every $n = 1, 2, \ldots, N$, then the artificial 'counting' population process will be almost surely unbounded as well. Indeed, if z_m is unbounded for a reaction m for which $\nu'_{N+1,m} > 0$, then this will result in an unbounded counting variable x_{N+1}, since the artificial 'counting' process can never be decremented. Thus, we must only show that, if z_m is unbounded for at least one reaction m for which $\nu'_{N+1,m} = 0$ and x_n is bounded for every $n = 1, 2, \ldots, N$, then x_{N+1} will also be unbounded.

Suppose there exists a DA z_m that is unbounded for a reaction m for which $\nu'_{N+1,m} = 0$ and that x_n is bounded, for every $n = 1, 2, \ldots, N+1$. Since $\nu'_{N+1,m} = 0$, we have that $\sum_{n=1}^{N} s_{nm} > 0$, by virtue of (S.18). Thus, reaction m increments the value of $\sum_{n=1}^{N} x_n$ an unbounded number of times. Since, for every $n = 1, 2, \ldots, N$, x_n is assumed to be bounded, this means that at least one reaction m' must decrement the value of $\sum_{n=1}^{N} x_n$ an unbounded number of times. This implies that there exists an unbounded DA $z_{m'}$ for some reaction m' for which $\sum_{n=1}^{N} s_{nm'} < 0$ or, equivalently, $\nu'_{N+1,m'} = -\sum_{n=1}^{N} s_{nm'} > 0$, by virtue of (S.18). This implies that x_{N+1} is unbounded, which contradicts our initial assumption and concludes the proof.

References

1. Horn RA, Johnson CR (1985) Matrix Analysis. New York, NY: Cambridge University Press.

2. Horn RA, Johnson CR (1991) Topics in Matrix Analysis. New York, NY: Cambridge University Press.

3. Crank KN (1988) A method for approximating the probability functions of a Markov chain. J Appl Prob 25: 808–814.
